# The PLET (Portable Laparoscopic Endo-Trainer) study: a randomized controlled trial of home- versus hospital-based surgical training

**DOI:** 10.1007/s00423-024-03375-z

**Published:** 2024-06-13

**Authors:** Christoph Kuemmerli, Katja Linke, Diana Daume, Nicolas Germann, Ralph Peterli, Beat Müller-Stich, Jennifer M. Klasen

**Affiliations:** 1 Department of Visceral Surgery, Clarunis University Digestive Health Care Center Basel, Spitalstrasse 21, Basel, 4031 Switzerland; 2 Department of General Surgery, Lucerne Cantonal Hospital, Lucerne, Switzerland; 3 University of Basel, Basel, Switzerland

**Keywords:** Education during residency, Surgical training, Laparoscopic skills, General surgery, Laparoscopy trainer

## Abstract

**Purpose:**

The purpose of this study was to assess the effect of training with a personal, portable laparoscopic endo-trainer (PLET) on residents’ laparoscopic skills.

**Methods:**

The study took place at a tertiary-care academic university hospital in Switzerland. All participants were randomized to either a home- or hospital-based PLET training group, and surgical skill performance was assessed using five laparoscopic exercises. 24 surgical residents, 13 females and 11 males, were enrolled at any training stage. Nine residents completed the assessments. Endpoints consisted of subjective and objective assessment ratings as well as exercise time and qualitative data up to 12 weeks. The primary outcome was the difference in exercise time and secondary outcomes included performance scores as well as qualitative data.

**Results:**

The hospital-based training group performed exercises number 1, 3 and 4 faster at 12 weeks than at baseline (*p* = .003, < 0.001 and 0.024). Surgical skill performance was not statistically significantly different in any of the endpoints between the hospital- and home-based training groups at 12 weeks. Both the subjective and objective assessment ratings significantly improved in the hospital-based training group between baseline and 12 weeks (*p* = .006 and 0.003, respectively). There was no statistically significant improvement in exercise time as well as subjective and objective assessment ratings over time in the home-based training group. The qualitative data suggested that participants who were randomized to the hospital-based training group wished to have the PLET at home and vice versa. Several participants across groups lacked motivation because of their workload or time constraints, though most believed the COVID-19 pandemic had no influence on their motivation or the time they had for training.

**Conclusion:**

The PLET enhances laparoscopic surgical skills over time in a hospital-based training setting. In order to understand and optimize motivational factors, further research is needed.

**Trial registration:**

This trial was retrospectively registered on clinicaltrials.gov (NCT06301230).

**Supplementary Information:**

The online version contains supplementary material available at 10.1007/s00423-024-03375-z.

## Introduction

Due to the patient benefit, laparoscopic procedures are the gold standard for most abdominal operations [1, 2]. Acquiring laparoscopic surgical skills has been one of the pivots during surgical education [3]. The Halsted method of “see one, do one, teach one” often used and cited in the past is increasingly being replaced by more intensive instruction such as Peyton’s 4-step method to ensure better outcomes and patient safety [4]. More intensive training requires extensive resources and more frequent teaching interventions. High-quality, frequent laparoscopic skill training in a safe environment was shown to be crucial in mastering laparoscopic skills [5–7].

Nevertheless, residents lack training opportunities during clinical training given other duties. Working hour restrictions and increased administrative duties of surgical residents limit their time to train laparoscopic skills [7]. Moreover, residency programs face a broad range of challenges to organize surgical training, especially due to the constantly expanding surgical approaches, including open, laparoscopic, or robot-assisted operations.

In the operating room (OR), it is common that surgical residents perform laparoscopic procedures under the appropriate supervision of a more experienced surgeon. This remains challenging in the current environment. Due to a gradual change in the healthcare system and the ongoing global COVID-19 pandemic, causing the cancellation of elective surgeries, learning opportunities in laparoscopic surgery have further decreased [8]. Shorter patient duration of hospital stays and pressure to increase procedures in an outpatient setting also do not support teaching the skills, but rather justify surgery done by senior surgeons due to shorter operating times, resulting in lower OR costs [9]. Nevertheless, training of surgical skills remains indispensable and crucial to guarantee safe surgical treatments for patients in the future [5].

Skills labs have therefore become increasingly important to facilitate training opportunities for surgical residents [10]. Laparoscopic training on different types of models is already part of the curriculum at several hospitals worldwide. Previous studies have shown that training on different types of models improves surgical skills in the OR [11, 12]. However, rare opportunities to train in the hospital during working hours remain a problem for most surgical residents due to their high workload [13]. Training outside the hospital might be an option to further promote the development of laparoscopic skills. Especially during a pandemic where home office and limited social contact are desirable, a flexible portable model, which can also be used for home-based training, offers opportunities.

## Materials and methods

This study aimed to evaluate the impact of surgical resident training with the portable laparoscopic endo-trainer (PLET) at home or in the hospital. We hypothesize that with a PLET, laparoscopic skills can be trained at home with similar performance as in hospital-based training. Surgical residents working at the Clarunis, Department of Visceral Surgery, University Hospital and St. Clara Hospital Basel, Switzerland were randomly assigned to a home- or hospital-based training group using the PLET in a 1:1 ratio. Clarunis is a tertiary hospital at two sites, the University Hospital and the St. Clara Hospital, with one general surgery residency program. The PLET is light and easy to transport as it’s packed in a bag and consists of a foldable plastic box, which can be assembled in less than 30 s by means of simple handles. There are three accesses for the surgical instruments (ports) as well as a central recess over which a tablet with an integrated camera can be placed (Fig. A.1). Inside the trainer, there are five different training modules. Available exercises are pegboard transfer (Fig. A.2a), placement and passing of ligating loop (Fig. A.2b), pattern cutting and dissecting (Fig. A.2c), suturing and intracorporeal knotting (Fig. A.2d) and bead placement (Fig. A.2e). The following instruments were available to the study participants: a needle holder, grasper, scissors, and forceps.

In an introductory lecture, all participants were informed about the study procedures and had time to familiarize themselves with the PLET and all exercises. Two senior visceral surgeons (Katja Linke (KL) and Jennifer M. Klasen (JK)) were available for the residents at any time in case of questions through support by phone, email or in person. Instructional videos, which were stored on the PLET’s tablets, were available for each of the five exercises and could be viewed at any time. In the home-based training group, the PLET was taken home, and training was carried out during free time or on-call duty at home. During hospital-based training, the PLET was in the hospital and training was possible during or immediately before and after working hours.

The primary endpoint was the evaluation of the in-group and between-group difference in surgical skill performance from baseline to 12 weeks measured by the Objective Structured Assessment of Technical Skills (OSATS) which consists of seven items of Likert-type scale assessments and is a validated tool to assess surgical skills (Table A.1) [14]. The secondary endpoints consisted of exercise time measurement and qualitative data regarding in-group and between-group comparisons over time.

**Fig. 1:**
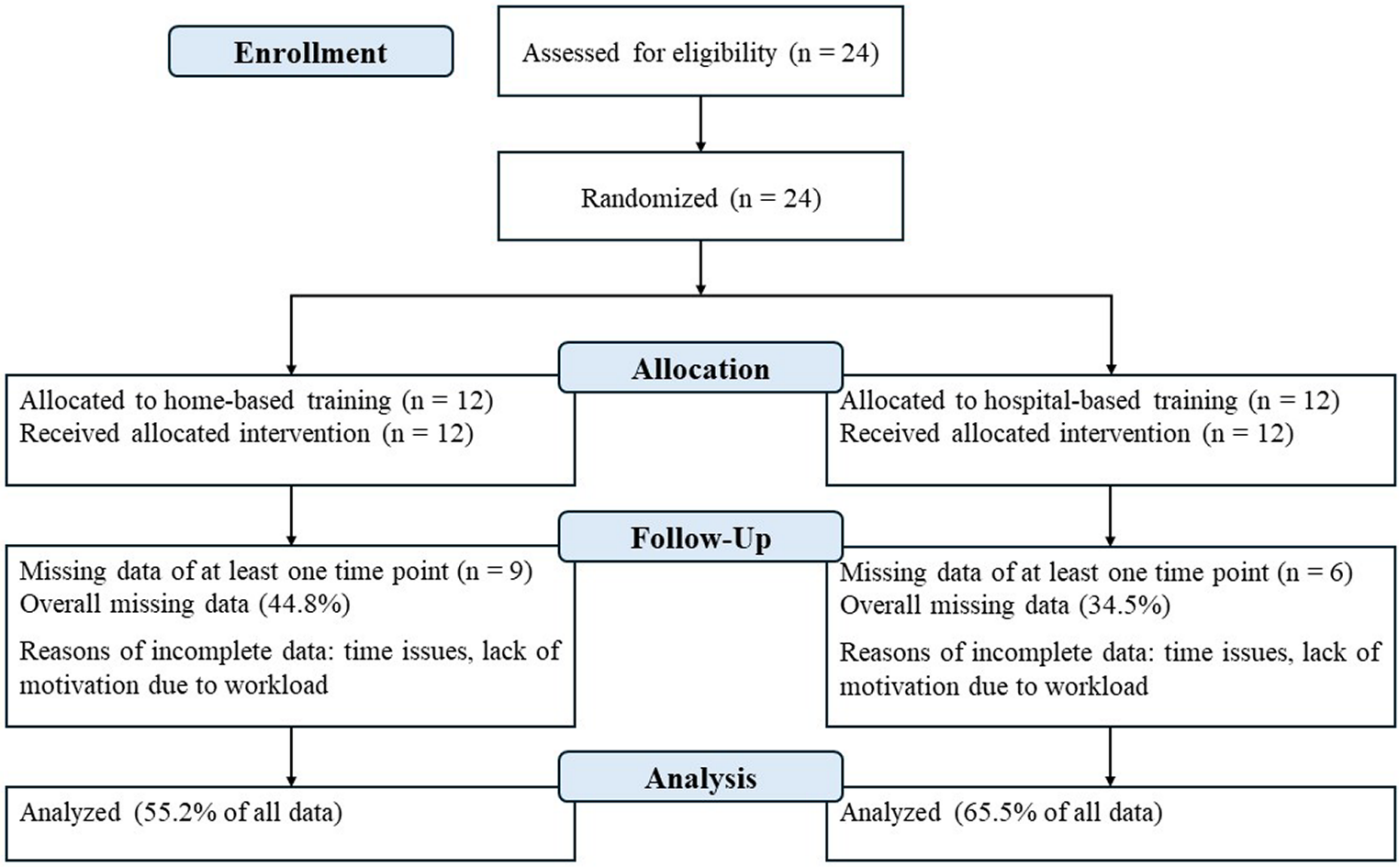
Flow diagram of participant recruitment

We collected qualitative data at the end of the study period to understand the challenges of the participants to fulfil the study requirements. The participants received open-ended questions via email and sent them back to the principal investigator. The questions focused on their motivation to participate in the study as well as their motivation to improve their surgical skills. The questionnaire also inquired about general and specific responses to the study and participation, as well as how the participants intend to improve their skills in the future and how the COVID-19 pandemic impacted motivation and time to train. Each participant received a previously prepared opaque envelope containing a black or white USB stick from the Principal Investigator with the assignments on it as part of the randomization process. The outcome assessors were blinded to the allocation. Figure 1 shows the flow diagram of participant recruitment.

The study design required a total of 28 participants for an expected 80% statistical power, while 24 participants resulted in the statistical power of 74% based on a power analysis with Gpower software for a within repeated-measures ANOVA (Analysis of Variance) with three measurement times and an expected medium effect size of Cohen’s d = 0.5 [15]. We performed statistical analysis using R Project for Statistical Computing [16]. All statistical models are based on a significance level of 5% (alpha = 0.05).

For descriptive purposes, we used the Shapiro-Wilk test for univariate normality of data, consisting of video-recording time and assessment rating. Conducting two-sample t-tests for independent samples, we then compared the video-recording time of the two groups (home- vs. hospital-based) and the assessment rating condition (subjective vs. objective) for each measurement at baseline, 6 weeks, and 12 weeks. As our data was not fully normally distributed as assessed graphically and by the Shapiro-Wilk test and Levene’s assumption test for homogeneity of variance was not satisfied in each case, we backed up statistically significant values of the *parametric* Student’s t-tests with non-parametric Mann-Whitney U tests [17]. To indicate the effect sizes of comparisons between two training conditions, we used Hedges g instead of Cohen’s d, since our sample size was small (< 50), and some data consisted of unequal variances [18].

We also conducted one-way (time) and mixed (time x training condition) repeated-measures ANOVA. To control random subject effects and other statistical biases, based on the unbalanced data distribution due to missing values, we created a mixed-effects model for repeated-measures ANOVA by using the lme4 package [19]. In the event data distribution failed to pass Mauchly’s assumption test of sphericity, the Greenhouse-Geisser (GG) method was used as GG-epsilon was > 0.75 in each case [20]. In this respect again, we used non-parametric Welch- and Kruskal-Wallis-tests as a backup for statistically significant parametric ANOVA results. Subsequently, Tukey’s post-hoc analysis showed relevant comparisons of statistically significant main and interaction effects [17]. For the qualitative data, we conducted a content analysis.

## Results

The median age of the 24 participants was 25.6 years and 12 (6 in both groups) were female. Nine were in postgraduate (PG) training year 1–2, 9 in PG year 3 or 5 and 6 in PG year 4 or higher (Table A.2). Nine residents had available data for all exercises and time, three in the home-based and six in the hospital-based training group. Overall, 139 of 252 data points were available in the home-based training group and 165 out of 252 in the hospital-based group.

**Fig. 2:**
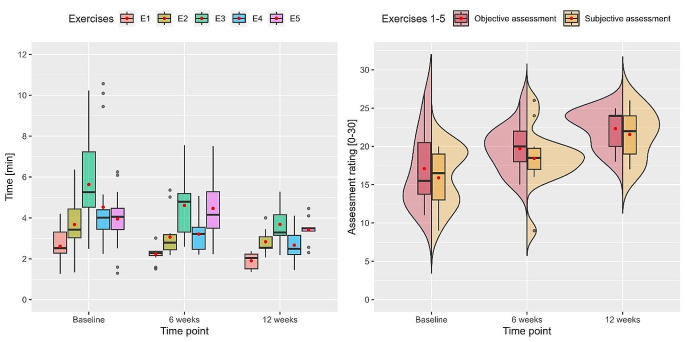
Comparison of time and assessment rating over time

**Fig. 3:**
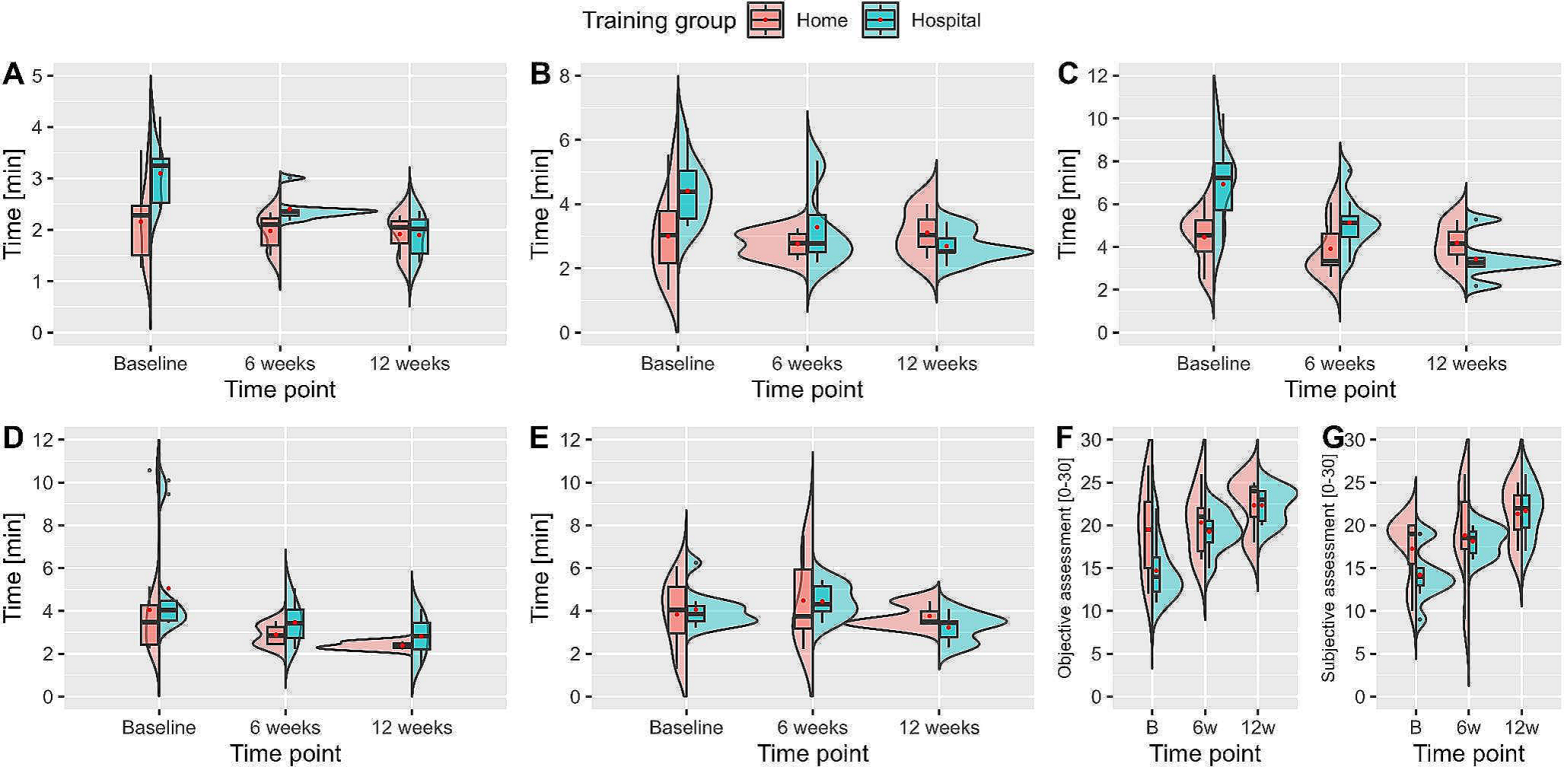
Comparison of exercise time for each of the five exercises (**A-E**) and the assessment rating (**F-G**)

Both the objective and subjective assessment ratings improved statistically significantly between the baseline and the 12-week time point of the hospital-based training group over all five exercises (*p* = .006 and 0.003). In contrast, there was not a statistically significant improvement in assessment rating in the home-based training group. However, participants with home-based training showed a slight trend towards better assessment ratings over the course of the study. As the normality assumption was violated in some cases, non-parametric tests consistently confirmed the parametric test results (Table 1 and A.3, Fig. 2). We found no statistically significant differences in the subjective and objective assessment ratings between the two training groups for each of the three measurement times (*p* = .389, 0.338, and 0.577) (Table A.4, Fig. 3F-G).

**Table 1:** Overall assessment score for both groups, home- and hospital-based training

	Exercises rating score (0–30) by assessment type and time point												
	Objective rating score mean ± SD						Subjective rating score mean ± SD						
	Home			Hospital			Home				Hospital		
**Exercises 1–5**	*BL*	*6 WK*	*12 WK*	*BL*	*6 WK*	*12 WK*	*BL*	*6 WK*	*12 WK*	*BL*		*6 WK*	*12 WK*
	19.50 ± 5.13	20.33 ± 3.88	22.33 ± 3.79	14.70 ± 3.37	19.25 ± 2.31	22.33 ± 1.97	17.27 ± 3.72	18.83 ± 5.98	21.33 ± 4.04	14.22 ± 3.19		18.13 ± 1.64	21.67 ± 3.27

SD; standard deviation, BL; baseline, WK; weeks

Participants of the hospital-based training group showed statistically significant improvements regarding exercise time over the three measurement times. There was no statistically significant improvement in exercise time in the home-based training group but a slight trend towards a faster performance (Table 2 and A.3, Fig. 2). There was a statistically significant difference in exercise time between home- and hospital-based training conditions for both the baseline and 6-week measurement times. Stratified by endpoint and exercise, the home-based training group performed faster than the hospital-based training group in exercises 1–3 from baseline (*p* = .004, 0.011, and 0.002) and in exercise 1 at the 6-week time point (*p* = .022). At 12 weeks, there was no statistically relevant difference in exercise time between home- and hospital-based training conditions (Table 2 and A.3, Fig. 3A-E).

**Table 2:** Exercise time for both groups, home- and hospital-based training

	Exercise time (in minutes) by training condition and time point									Home vs. hospital		
	Home time mean ± SD			Hospital time mean ± SD			Overall time mean ± SD			*p* (Two-sample ind. t-test) *p* (Mann-Whitney U test)		
**Exercise**	*Baseline*	*6 WK*	*12 WK*	*Baseline*	*6 WK*	*12 WK*	*Baseline*	*6 WK*	*12 WK*	*Baseline*	*6 WK*	*12 WK*
1) Pegboard transfer	2.16 ± 0.74	1.98 ± 0.35	1.92 ± 0.45	3.10 ± 0.58	2.41 ± 0.25	1.90 ± 0.43	2.61 ± 0.81	2.22 ± 0.36	1.91 ± 0.40	0.004** 0.005**	0.022* 0.012*	0.955
2) Placement and passing of ligating loop	3.00 ± 1.28	2.76 ± 0.41	3.12 ± 0.85	4.42 ± 0.99	3.28 ± 1.22	2.69 ± 0.49	3.68 ± 1.34	3.06 ± 0.97	2.83 ± 0.61	0.011* 0.015*	0.339	0.360
3) Pattern cutting and dissecting	4.46 ± 1.23	3.91 ± 1.34	4.19 ± 1.06	6.93 ± 1.87	5.14 ± 1.29	3.43 ± 1.02	5.64 ± 1.98	4.62 ± 1.41	3.68 ± 1.04	0.002** 0.005**	0.108	0.333
4) Suturing and intracorporal knotting	4.05 ± 2.37	2.90 ± 0.50	2.38 ± 0.20	5.05 ± 2.52	3.47 ± 1.02	2.81 ± 0.98	4.53 ± 2.44	3.21 ± 0.85	2.67 ± 0.81	0.359	0.233	0.345
5) Bead placement	3.84 ± 1.56	4.48 ± 2.10	3.77 ± 0.60	4.07 ± 0.87	4.45 ± 0.77	3.23 ± 0.67	3.95 ± 1.25	4.46 ± 1.42	3.41 ± 0.67	0.681	0.970	0.282

SD; standard deviation, WK; weeks

The post-interventional qualitative data confirmed that most participants admitted that they could have trained harder. However, they often lacked motivation due to workload, time issues and much needed recovery time. Most participants felt that the circumstances around the COVID-19 pandemic did not influence their motivation or the time they had for training. Participants who were randomized to hospital-based training wished to have the surgical trainer at home and vice versa.

## Discussion

This study showed that the use of a PLET improves the subjective and objective quality of surgical performance as well as speed [13, 21]. These findings were statistically significant in the hospital-based training group, and we observed a slight trend towards improvement for the home-based condition. Although at the beginning of the study, the hospital-based training group started with faster performance than their counterparts who trained at home, there were no differences at the 12-week time point between the two groups. The fact that participants had faster baseline performance at the hospital than at home may be due to the well-known training environment as it took place during their daily work routine. A slower performance in the home-based training group at baseline left more room for improvement over the course of the study, making it likely that participants performed faster comparatively.

However, the motivation to train was poor in both groups. At the beginning of the study, all participants were very excited about the opportunity to participate and the PLETs were highly valued. As they progressed though, many became less motivated to train and to participate, despite intensive support efforts on the part of the senior visceral surgeons and recognition of their own progress. Participants understood they should train more, but what ultimately prevented them from doing so? Very long working hours or being overworked reduce intrinsic motivation, as stated by some participants. The desire for part-time work among surgeons shows the importance of addressing and mitigating this problem [22]. Extrinsic incentives can improve training intensity and thus progress [23]. These incentives could include rewards, competitions, and conditions that require succeeding at certain levels of exercises before being allowed to operate in the OR [23]. Exploring further methods to enhance motivation and training effectiveness seems necessary. The results of this study suggest that improving performance and better patient outcomes is not enough motivation for residents to train more, which seems to be strange, especially because participants are seen as highly motivated in their day-to-day clinical work. Mandatory training requirements and testing may improve uptake. Perhaps a competition between participants including immediate feedback or some form of reward could increase motivation and perseverance [24, 25]. Other authors recommend mandatory completion of a training program before active participation in the OR. A faster time of implementation with qualitative improvement has been demonstrated using this approach [26]. Gostlow et al. describe intrinsic motivational factors such as unlimited access to the trainer or simulator, including training during work hours if possible. The authors further described the setting of performance goals, competition, and assurance of implementation in clinical practice such as using the learned skills in the OR. They further included extrinsic motivational factors such as the possibility of training during work hours with extra time reserved for this purpose, easy and close access to the trainer, having a dedicated quiet space to use trainer, the possibility of contacting an instructor and mandatory implementation [27]. Other forms of training may be of interest. For example, training in pairs (known as dyad training) reduce training time, which may offer an advantage when training time is limited [28]. Artificial intelligence (AI) may become important in surgical training. Computer vision, augmented reality, and artificial intelligence algorithms have already been described for an online laparoscopic surgical skills assessment [29]. Also, AI was used to assess laparoscopic simulation videos regarding motion metrics [30].

Another consideration is whether a 12-week model is too long, leading to motivation decreasing over time. It may make more sense to offer shorter training periods, especially because we have seen a decrease in cooperation (videos returned) over time. On the other hand, there have been studies of programs over a much longer period but without reporting compliance [31].

This study also showed that the participants’ self-assessment, including their improvement, matched the objective assessment. This demonstrates that the training was effective even without constant supervision. Even if the results were not as good as in the group that trained at the hospital, during the COVID-19 pandemic, the PLET was a very useful tool to train laparoscopic skills at home given that time in the hospital had to be held as short as possible to reduce social contacts and thus the risk of infection. Training on the PLET certainly cannot fully replace operating on a patient under supervision, as confirmed by Crochet et al. in the context of a performance gap in the transfer from the trainer to the real patient [32]. However, a foundation can be laid, increasing patient safety, and reducing the time required in the OR through the use of a fairly inexpensive trainer.

Due to missing values and only nine participants completing the data set and videotaping each exercise, statistical power was low. Therefore, the results should be interpreted with caution. Nevertheless, all statistical analyses showed a trend towards improvement in laparoscopic skills regarding exercise time and assessment rating over the course of the study. Moreover, the residents in the hospital-based group were more experienced. However, residents in the hospital-based group had neither higher baseline assessment scores nor performed the exercises faster suggesting that this difference in experience is not relevant in this study.

## Conclusion

A personal laparoscopic training device improves laparoscopic surgical skills over time. With its usage, residents can even significantly improve their performance in basic surgical skills. While it does appear to make a difference whether they have the trainer at home or in the hospital at the start of the training period, there seems to be no relevance of this condition at the end of the training period. Furthermore, our study results suggest that surgical training should be done during working hours.

The study raises the question of why residents who are highly motivated in their daily work showed such low motivation to learn from the trainer. At the beginning of the study, all participants were very excited about the opportunity to participate and the PLETs were in high demand. However, as participants progressed, many of them became less motivated to train and to participate, despite intensive support efforts and recognition of their own progress. Improving one’s own laparoscopic skills does not seem to be sufficiently motivating, possibly due to an already stressful workday due to long work hours. Further research is needed to understand and optimize the motivational factors of residents to identify the laparoscopic skills training setting.

### Electronic supplementary material

Below is the link to the electronic supplementary material.

Supplementary Material 1

Supplementary Material 2

Supplementary Material 3

## Data Availability

No datasets were generated or analysed during the current study.

## Abbreviations

AI: Artificial Intelligence
GG: Greenhouse-Geisser
OR: Operating Room
OSATS: Objective Structured Assessment of Technical Skills
PG: Postgraduate
PLET: Portable Laparoscopic Endo-Trainer
