# Supplementary material for: The PLET (Portable Laparoscopic Endo-Trainer) study: a randomized controlled trial of home- versus hospital-based surgical training
Source: Langenbecks Arch Surg. 2024 Jun 13;409(1):186. doi: 10.1007/s00423-024-03375-z (PMC11176216; doi:10.1007/s00423-024-03375-z)
Supplement: Supplementary file 1 — Supplementary Material 1 [file 423_2024_3375_MOESM1_ESM.docx]

### **Appendix**

**Table A.1.** Assessment and grading for each of the five exercises.

| Exercise | Description | 5-point rating scale (1 = worst; 5 = best performance) |
| --- | --- | --- |
| 1) Suturing and knot tying | - Needle positioning - Knotting technique - flow (linear and rotational movements) - precision (quality of knot) | 1-5 |
| 2) Peg transfer | - flow (linear and rotational movements) - precision (transfer of peg and peg placement) | 1-5 |
| 3) Cutting and dissecting | - Instrument handling - flow (linear and rotational movements) - precision (shape of the cut circle) | 1-5 |
| 4) Bead placement | - flow (linear and rotational movements) - precision (placement and removal of beads) | 1-5 |
| 5) Thread passing | - flow (linear and rotational movements) - Precision of movement (threading) | 1-5 |

**Table A.2.** Sex and experience of study participants.

|  | | **Home-based group**  **(N = 12)** | **Hospital-based group**  **(N = 12)** |
| --- | --- | --- | --- |
| Sex, n (%) | |  |  |
|  | Female | 6 (50) | 6 (50) |
|  | Male | 6 (50) | 6 (50) |
| PG expertise level, n (%) | |  |  |
|  | Beginner (PGY 1/2) | 6 | 3 |
|  | Moderate (PGY 3/4) | 4 | 5 |
|  | Advanced (PGY 5/6) | 2 | 4 |

**Table A.3.** Comparison of time and rating score stratified by exercise.

|  | | | *Analysis of Variance* | | | | | | |  | | *Tukey’s HSD post hoc tests* | | | | | | |  |
| --- | --- | --- | --- | --- | --- | --- | --- | --- | --- | --- | --- | --- | --- | --- | --- | --- | --- | --- | --- |
|  | | | *Sphericity assumption (Mauchly)* | | |  | | | |  | |  | | | | | | |  |
|  | | |  |  |  | *One-way and mixed repeated-measures ANOVA* | | | |  | | *p adj.* | | | | | | |  |
|  | | | *W* | *p* | *GGε*  *GGp* | *F* | *df* | *p*  *Welch p / Kruskal-Wallis p* | *η2* | |  | | *Baseline vs. 6 weeks* | | *Baseline vs. 12 weeks* | | *6 weeks vs. 12 weeks* | | |
| **Exercise** | | |  | | | | | | |  | |  | |  | |  | | |  |
|  | **Pegboard transfer** | |  | | |  |  |  |  | |  | |  | |  | |  | | |
|  | | Time | .804 | .521 |  | 3.980 | 2 | .027*  .022* / .014* | .17 | |  | | .197 | | .029* | | .501 | | |
|  | | Time x training condition | .877 | .721 |  | 2.482 | 2 | .097 | .07 | |  | |  | | Hospital  .003** | |  | | |
|  | **Placement and passing of ligating loop** | |  | | |  |  |  |  | |  | |  | |  | |  | | |
|  | | Time | .770 | .401 |  | 2.296 | 2 | .114 | .10 | |  | | .258 | | .154 | | .883 | | |
|  | | Time x training condition | .614 | .232 |  | 2.572 | 2 | .090 | .09 | |  | |  | |  | |  | | |
|  | **Pattern cutting and dissecting** | |  | | |  |  |  |  | |  | |  | |  | |  | | |
|  | | Time | .517 | .099 |  | 4.691 | 2 | .015*  .007** / .024* | .19 | |  | | .188 | | .014* | | .396 | | |
|  | | Time x training condition | .363 | .048* | .611  .114 | 3.942 | 2 | .028*  .009** / .001** | .11 | |  | |  | | Hospital  <.001*** | |  | | |
|  | **Suturing and intracorporal knotting** | |  | | |  |  |  |  | |  | |  | |  | |  | | |
|  | | Time | .326 | .035* | .597  .043* | 4.077 | 2 | .024*  .012* / .009** | .17 | |  | | .113 | | .037* | | .775 | | |
|  | | Time x training condition | .347 | .071 |  | .094 | 2 | .911 | <.01 | |  | |  | | Hospital  .024* | |  | | |
|  | **Bead placement** | |  | | |  |  |  |  | |  | |  | |  | |  | | |
|  | | Time | .979 | .929 |  | 2.089 | 2 | .136 | .09 | |  | | .442 | | .517 | | .119 | | |
|  | | Time x training condition | .762 | .442 |  | .280 | 2 | .765 | .01 | |  | |  | |  | |  | | |
| **Assessment** | | |  | | | | | | |  | |  | |  | |  | | |  |
|  | **Subjective** | |  | | |  |  |  |  | |  | |  | |  | |  | | |
|  | | Time | .762 | .387 |  | 7.405 | 2 | .002**  .002** / .009** | .27 | |  | | .137 | | .001** | | .133 | | |
|  | | Time x training condition | .869 | .655 |  | 3.540 | 2 | .047* | .03 | |  | |  | | Hospital  .006** | |  | | |
|  | **Objective** | |  | | |  |  |  |  | |  | |  | |  | |  | | |
|  | | Time | .310 | .030* | .592  .004** | 5.795 | 2 | .006**  .003** / .008** | .22 | |  | | .150 | | .005** | | .275 | | |
|  | | Time x training condition | .342 | .069 |  | 1.322 | 2 | .291 | .06 | |  | |  | | Hospital  .003** | |  | | |
| GG; Greenhouse-Geisser method | | | | | | | | | | | | | | | | | |  |  |

**Table A.4.** Assessment rating, objective vs. subjective.

|  |  |  | | *Two-sample t-test (independent) with Levene’s assumption test* | | | | | | | | | | | | | | | |  |  |  |
| --- | --- | --- | --- | --- | --- | --- | --- | --- | --- | --- | --- | --- | --- | --- | --- | --- | --- | --- | --- | --- | --- | --- |
|  | *Objective vs. subjective assessment rating* | | | | | | | | | | | | | | | | | | | | | |
|  | *Baseline* | | | | | | |  | *6 weeks* | | | | |  | *12 weeks* | | | | | | | |
|  | *Levene p* | | *t* | | *df* | *t-test*  *p* | *Hedges' g* |  | *Levene p* | *t* | *df* | *t-test*  *p* | *Hedges' g* |  | | *Levene p* | *t* | *df* | *t-test*  *p* | | *Hedges' g* |  |
| **Exercises**  **1-5** | .353 | | -.87 | | 38 | .389 | .270 |  | .757 | -.98 | 26 | .338 | .358 |  | | .594 | -.57 | 16 | .577 | | .256 |  |
