# Supplementary figures and images for: The PLET (Portable Laparoscopic Endo-Trainer) study: a randomized controlled trial of home- versus hospital-based surgical training
Source: Langenbecks Arch Surg. 2024 Jun 13;409(1):186. doi: 10.1007/s00423-024-03375-z (PMC11176216; doi:10.1007/s00423-024-03375-z)

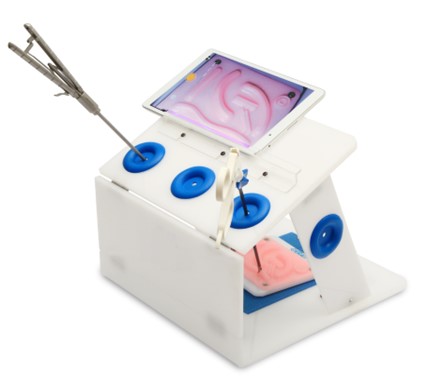

Supplement: Supplementary file 2 — Supplementary Material 2 [file 423_2024_3375_MOESM2_ESM.jpg]

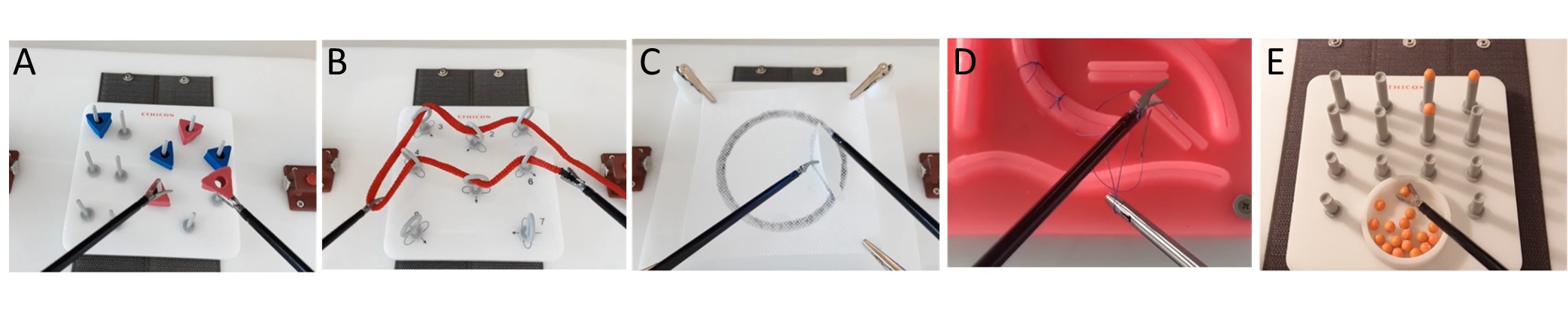

Supplement: Supplementary file 3 — Supplementary Material 3 [file 423_2024_3375_MOESM3_ESM.jpg]
